# A Review of Crucial Radiological Investigations in the Management of COVID-19 Cases

**DOI:** 10.7759/cureus.36825

**Published:** 2023-03-28

**Authors:** Mathangi Rajaram-Gilkes, Hamzah Shariff, Nevin Adamski, Sophia Costan, Marybeth Taglieri, Marios Loukas, R. Shane Tubbs

**Affiliations:** 1 Medical Education, Geisinger Commonwealth School of Medicine, Scranton, USA; 2 Anatomical Sciences, St. George's University, St. George, GRD; 3 Neurosurgery/Structural & Cellular Biology, Tulane University School of Medicine, New Orleans, USA

**Keywords:** rt-pcr, radiological findings, consolidation, ground-glass opacities, sars-cov-2, viral pneumonia, ultrasound, x-ray, ct, covid-19

## Abstract

Chest X-ray, chest CT, and lung ultrasound are the most common radiological interventions used in the diagnosis and management of coronavirus disease 2019 (COVID-19) patients. The purpose of this literature review, which was performed according to Preferred Reporting Items for Systematic Reviews and Meta-Analyses (PRISMA) guidelines, is to determine which radiological investigation is crucial for that purpose. PubMed, Medline, American Journal of Radiology (AJR), Public Library of Science (PLOS), Elsevier, National Center for Biotechnology Information (NCBI), and ScienceDirect were explored. Seventy-two articles were reviewed for potential inclusion, including 50 discussing chest CT, 15 discussing chest X-ray, five discussing lung ultrasound, and two discussing COVID-19 epidemiology. The reported sensitivities and specificities for chest CT ranged from 64 to 98% and 25 to 88%, respectively. The reported sensitivities and specificities for chest X-rays ranged from 33 to 89% and 11.1 to 88.9%, respectively. The reported sensitivities and specificities for lung ultrasound ranged from 93 to 96.8% and 21.3 to 95%, respectively. The most common findings on chest CT include ground glass opacities and consolidation. The most common findings on chest X-rays include opacities, consolidation, and pleural effusion. The data indicate that chest CT is the most effective radiological tool for the diagnosis and management of COVID-19 patients. The authors support the continued use of reverse transcription polymerase chain reaction (RT-PCR), along with physical examination and contact history, for such diagnosis. Chest CT could be more appropriate in emergency situations when quick triage of patients is necessary before RT-PCR results are available. CT can also be used to visualize the progression of COVID-19 pneumonia and to identify potential false positive RT-PCR results. Chest X-ray and lung ultrasound are acceptable in situations where chest CT is unavailable or contraindicated.

## Introduction and background

In December 2019, a novel respiratory disease began to infect communities in Wuhan City, Hubei Province, China. On February 11, 2020, the causal virus was officially named coronavirus disease 2019 (COVID-19). According to Gaia et al. [1], the World Health Organization (WHO) categorized COVID-19 as a pandemic on March 11, 2020. As of July 7, 2021, the WHO reports over 180,000,000 cases and over 3,900,000 deaths worldwide [2].

Abbasi-Oshaghi et al. [3] state that although the initial source of COVID-19 is currently unknown, it is believed to have been transmitted by bats, vipers, or pangolins. A study by Sharma et al. [4] analyzed the RNA sequences of several animal coronaviruses and tentatively concluded that there could have been recombination between bat and pangolin coronaviruses before transmission to humans occurred. Though the above authors [3,4] express different sources of the disease in their articles, based on ongoing assessments by the U.S. Department of Energy, a definitive origin is yet to be determined.

The virus can be transferred between different types of mammals and birds and is also highly contagious among humans. Some of those infected with COVID-19 are asymptomatic and others develop a cough, shortness of breath, or fever. The virus is considered airborne as it is transmitted between humans via air droplets resulting from coughing or sneezing. It can also be spread via objects contaminated with respiratory droplets.

Considering the rapid spread of this disease, and the overwhelming volume of hospitalized COVID-19 patients worldwide, it is important to consider methods of diagnosis and management and how these methods can potentially be used to manage patient care and predict outcomes.

Currently, reverse transcription polymerase chain reaction (RT-PCR) remains the most cost-effective method for the initial diagnosis of COVID-19. A Hong Kong study by Kaufman et al. [5] analyzed 64 cases of positive COVID-19 infection and found that RT-PCR had a much higher sensitivity to early disease than chest X-ray. Throughout the progression of this pandemic, RT-PCR has remained the gold standard for diagnosis. Despite its many advantages in the diagnosis of COVID-19, it also has some limitations. For instance, Carpenter et al. & Chamorro et al. state that several studies have shown that RT-PCR has a high false negative rate [6,7]. According to Waller et al. [8] other scientists have cited disadvantages such as low sensitivity, and low clinical utility owing to a shortage of test kits in certain regions. The sensitivity of RT-PCR can also vary quite dramatically depending on the type of swab. Machnicki et al. [9] state that bronchoalveolar lavage is reported to have the highest sensitivity of 93%; nose and mouth swabs have a sensitivity of 71%, and throat swabs have the lowest sensitivity of 32%.

Throughout the past year, various radiological techniques have frequently been discussed in the conversation surrounding COVID-19 diagnosis, management, and outcome prediction; computed tomography (CT) and X-ray (radiographs) have been compared for their value in diagnosing and choosing management plans for COVID-19 cases.

**Figure 1:**
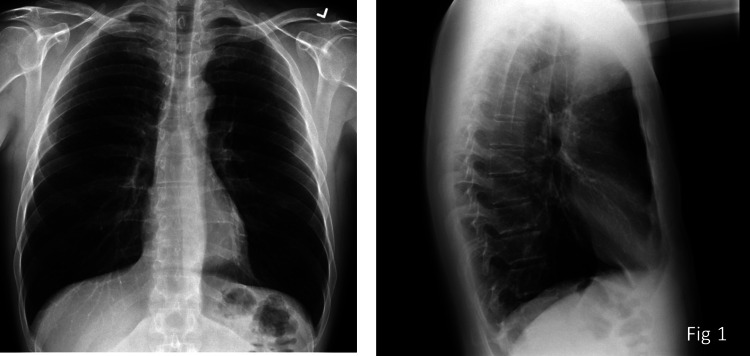
X-ray images of healthy chest and lungs AP view (left-hand side image) and lateral (right-hand side image) views. Image source: Reference no. [10]

Chest X-ray is very commonly used for diagnostic imaging of patients suspected of having COVID-19. Images of normal chest X-rays (Gaillard F, 2021) are displayed below [10] for reference and comparison (Figure 1). The image on the left shows the anterior-posterior (AP) view of the chest and the image on the right shows the lateral view of an essentially normal chest X-ray of a 50-year-old male revealing lungs with normal bronchovascular markings and well-defined tracheal passage. There is a degree of hyperinflation as evidenced by both increased retrosternal airspace and somewhat flattened and depressed diaphragms.

However, owing to the low sensitivity of chest X-rays in less severe cases, and the high likelihood that no abnormalities are displayed in COVID-19 patients with initial symptoms, X-ray is not recommended as the preferred imaging technique for COVID-19 diagnosis. Aljondi and Alghamdi (2020) report that lung ultrasound (LUS) can be used in the diagnosis and eventual management of COVID-19, especially when modalities such as chest CT are contraindicated (e.g., in pregnancy or chronic renal disease) [11]. Kalra et al. [12] report that when indicated, a non-contrast chest CT is generally performed at the lowest dose to minimize imaging artifacts and radiation exposure.

**Figure 2:**
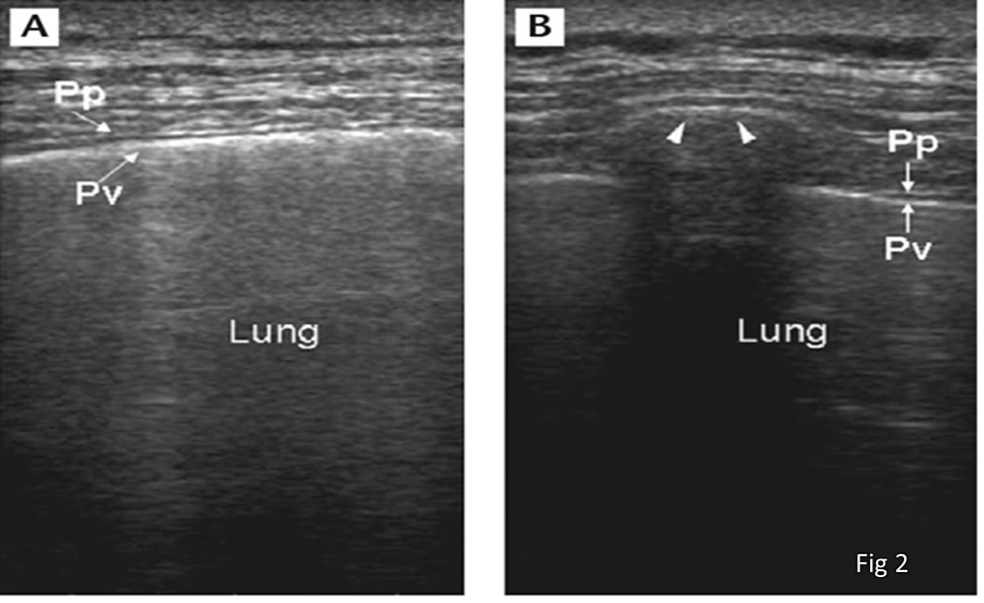
Normal chest ultrasound images (a) transverse scan of intercostal space; (b) longitudinal scan along the ribs. Pp= parietal pleura; Pv=visceral pleura. Image source: Reference no. [13]

The advantages of LUS include portability and cost-effectiveness. Normal LUS images are displayed below in (Figure 2). In image A the lung parenchyma can be visualized via a window between the ribs. The parietal pleura Pp and visceral pleura Pv can be identified by the arrows. In image B, shadow of a rib can be seen at the arrowheads and the parietal and visceral pleura can be identified at the arrows. 

**Figure 3:**
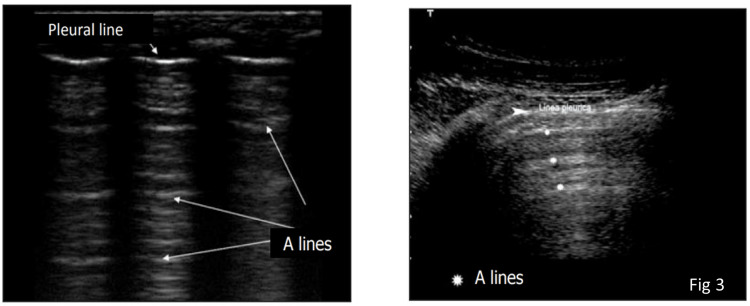
Lung ultrasound images “A” lines on LUS indicate a healthy lung. ”A” lines are horizontal white lines underneath the pleural line. Image source: Reference no. [13]

In Figure 3, the image on the left indicates a pleural line at the short arrow and 'A' lines by long pointers. Image on the right shows another example of 'A' lines represented with multiple asterisks, found beneath the pleural line (arrowhead). The pleural line is the labeled hyperechoic line that represents the junction of the visceral and the parietal pleura. The A-line artifacts are clearly visualized as horizontal reverberation artifacts of the hyperechoic pleural line. The rib shadows separate the intercostal spaces.

Because the range of reported sensitivity and specificity values is wide, and comprehensive guidelines for ultrasound use in COVID-19 are lacking, the true worth of ultrasound in management of this disease is not clear. However, chest CT is much more valuable than other modalities for the diagnosis and management of COVID-19 patients. CT imaging is very useful for distinguishing healthy, non-diseased lungs from lungs infected with COVID-19.

**Figure 4:**
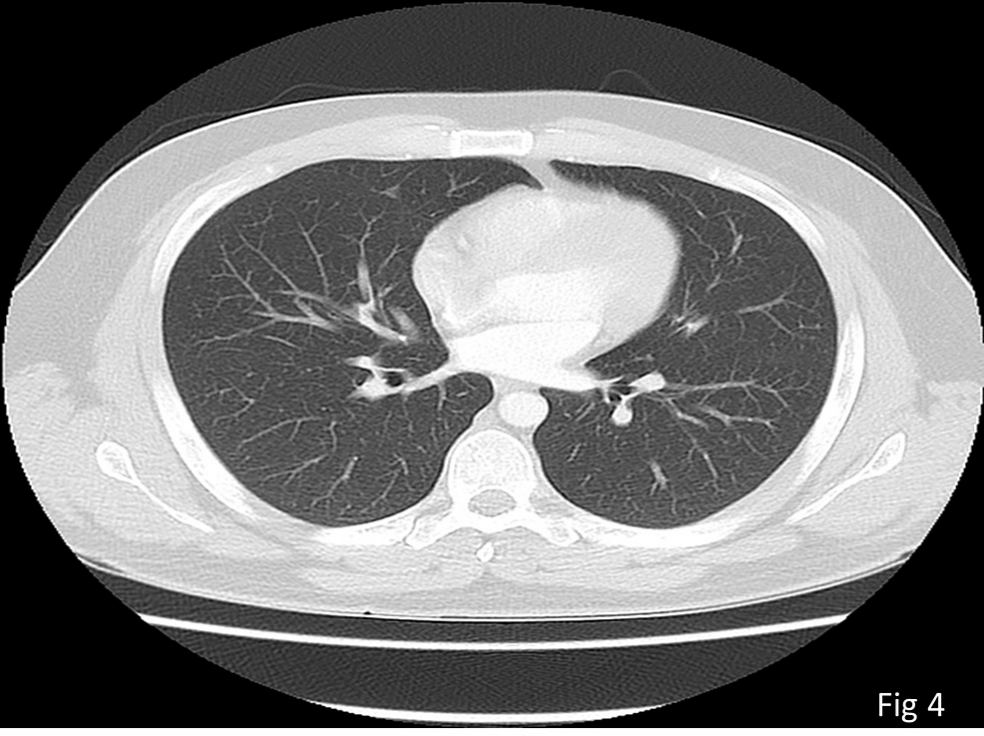
CT image of a healthy lung CT image of healthy lungs showing normal CT ratio. Normal bronchovascular markings can be visualized. The image is adapted from radiopedia.org (free access). Image source: Reference no. [12]

CT imaging is very useful for distinguishing healthy, non-diseased lungs from lungs infected with COVID-19. Kalra et al. report that when indicated, a non-contrast chest CT is generally performed at the lowest dose to minimize imaging artifacts and radiation exposure [12]. In the CT image below (Figure 4), which is adapted from radiopedia.org (free access), a section of the chest is taken at the T4 vertebral level. The lung fields are clear with normal bronchovascular markings. The heart is in middle mediastinum and of normal size.

On CT, patients with COVID-19 usually show several common characteristics. Most frequent in the early stages of infection is ground-glass opacity (GGO), a region of increased attenuation (or brightness) that also contains intact bronchial and vascular markings. GGO can also appear in diseases and infections other than COVID-19 [13].

Another major CT finding is lung consolidation, where the alveolar spaces of the lung that is normally filled with air can be occupied instead with some other type of fluid. Although consolidation is not always present in the early stages of GGO, it is likely to be present in later stages when more lung injury is expected. Finally, a reticular pattern (when interlobular septa or intralobular lines begin to thicken) and interlobular septal thickening can be another common characteristic finding in CT. Ye et al. discuss that these findings are thought to potentially result from lymphocyte invasion of the lungs’ interstitial spaces [14].

CT presentation is often quite varied and complicated, as not all suspected patients present with the above findings, and some present with additional findings such as lung fibrosis. Depending on disease progression, patients can also display a feature called crazy paving, a pattern in which interlobular septal thickening overlaps with GGO [7].

Several severe complications can occur in COVID-19 patients, which can impede proper healing and lead to disability or death. One such complication is acute respiratory distress syndrome (ARDS), characterized by an extensive release of inflammatory cytokines that can result in hypoxemia and pulmonary edema. This syndrome can be visualized on chest CT through peripheral GGOs. Patients with COVID-19 are also susceptible to superimposed pneumonia, which can be visualized as lobar consolidation on chest CT. Finally, patients can develop pulmonary embolism, which increases the risk of death immensely. The virus possibly activates the coagulation cascade, although the exact mechanism is not known. On chest CT, a pulmonary embolism due to COVID-19 can present as bilateral diffuse GGO, consolidation in both lungs and bronchial dilation [15].

Mingli et al. [16] reviewed several articles which indicate that chest CT is extremely useful for visualizing pulmonary findings such as GGO and consolidation in COVID-19, and some have used a quantitative COVID-19 Reporting and Data System (CO-RADS) score that could predict the likelihood of COVID-19 infection based on specific radiological findings.

The aims of this study are to emphasize the importance of radiology in visualizing many of the pulmonological findings in COVID-19 patients, and to determine how these images are integrated into the diagnosis and management of COVID-19 patients.

During this historic pandemic, it is incredibly important to identify specific methods and techniques for diagnosing the disease and assessing its severity. If physicians, healthcare staff, and researchers can identify key pulmonary features on chest CT in detail, quickly and accurately, they will be able to determine disease severity, prevent diagnostic error, direct patients to proper treatment and resources, and ultimately save countless lives.

## Review

Methods

This study was a systematic review without meta-analysis, conducted according to the Preferred Reporting Items for Systematic reviews and Meta-Analyses (PRISMA) statement. Information was sourced from the databases PubMed, Medline, AJR, PLOS, Elsevier, NCBI, and ScienceDirect. Key search words included COVID-19, CT, management, X-ray, ultrasound, infectious disease, viral pneumonia, diagnosis, Coronavirus-19, SARS-Cov-2, Ground-glass opacities, consolidation, radiological findings, and RT-PCR. The search period ranged from July-August 2021 and data extraction was limited to articles published towards the beginning of the COVID-19 pandemic, from January 1, 2020 to August 6, 2020.

**Figure 5:**
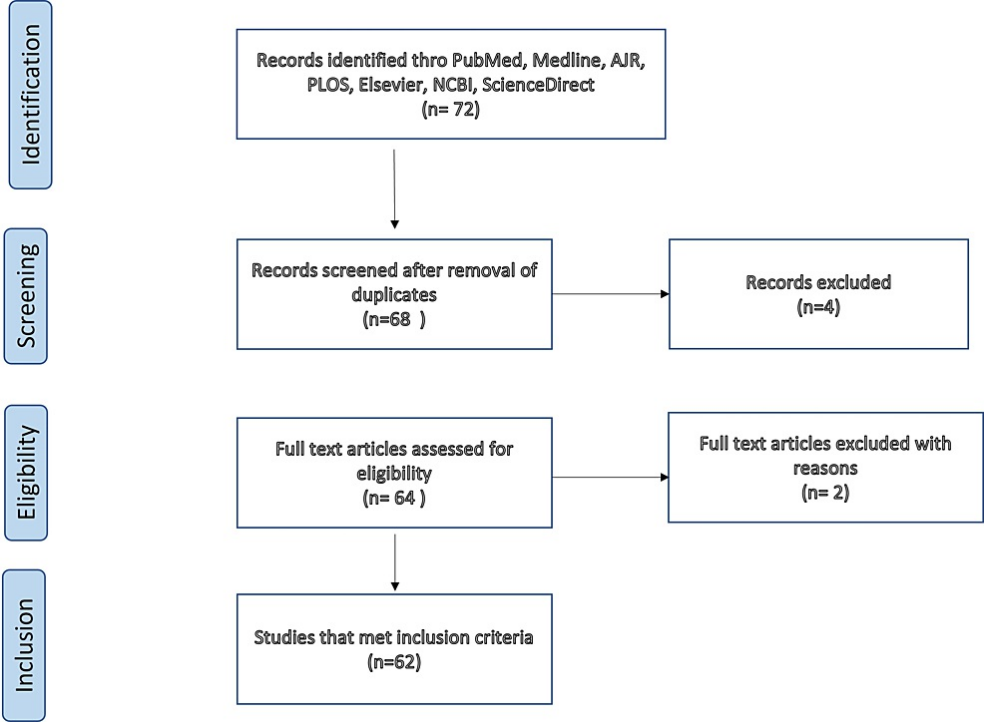
PRISMA flow chart of literature review. PRISMA: Preferred Reporting Items for Systematic Reviews and Meta-Analyses.

In total, 72 articles were analyzed for information about radiological findings in COVID-19 patients and the use of various radiological interventions in the diagnosis and management of such patients. They included 50 articles discussing normal and abnormal chest CT findings, 15 discussing chest X-ray findings, five discussing lung ultrasound appearances, and two discussing COVID-19 epidemiology, all these being the inclusion criteria. The articles were organized on a spreadsheet and the authors collaborated on brief descriptions for each article. The descriptions included the type of article, statistics cited, strengths and weaknesses, number of times cited, relevant conclusions, and helpful images. Normal and abnormal images for each radiological intervention were organized into a separate image database. Articles and image websites that contained duplicate information were removed. A search flow diagram is provided in Figure 5.

Results

CT Imaging in the Diagnosis and Visual Assessment of COVID-19

In recent systematic reviews and the primary literature, computed tomography (CT) has been closely studied as a supplemental and even a standard method in the clinical diagnosis and management of COVID-19. Arguably, Li M [17] states that the variation in diagnostic sensitivity of RT-PCR is a shortcoming for which CT could compensate. One study by Fang et al. [18] of 51 patients determined the sensitivity of CT to be significantly greater than that of initial RT-PCR (98% vs. 71%, respectively). In another study of 888 patients by Ai et al. [19], the sensitivity of CT was 97%, but the specificity was only 25%. A meta-analysis by Duarte et al. [20] of 168 studies that totaled 1,045 CT scans found the sensitivity of CT to be 95.3%. However, in a retrospective study of 568 patients by Salehi-Pourmehr et al. [21] with suspected COVID-19, CT imaging yielded a lower sensitivity of 64% but a specificity of approximately 77%.

**Figure 6:**
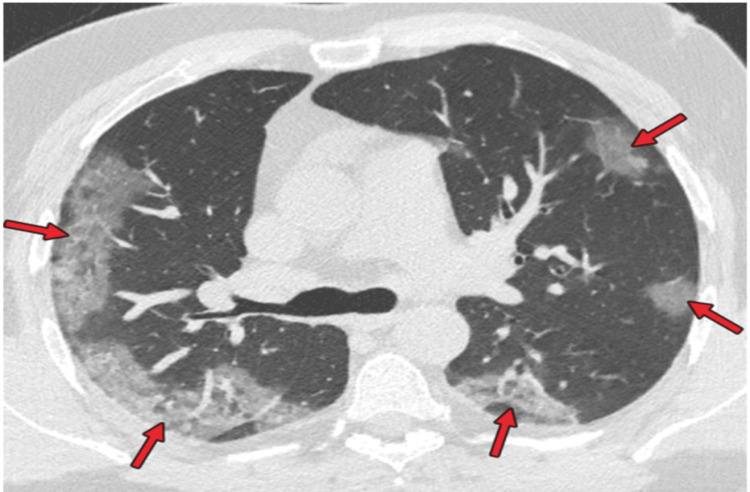
Axial non-enhanced chest CT image The image shows the bilateral and peripheral distribution of ground-glass opacities (GGOs) (arrows) in COVID-19 pneumonia. Image source: Reference no. [15]

**Figure 7:**
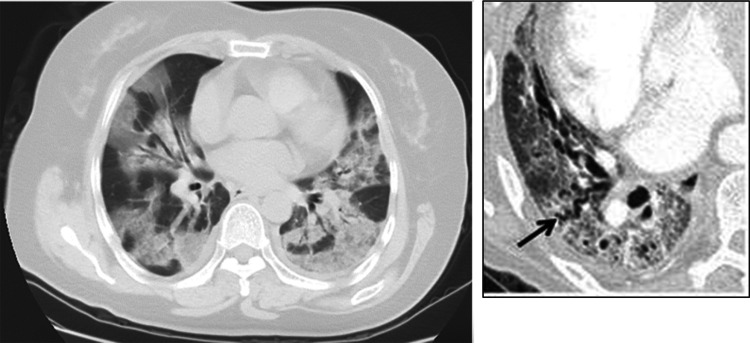
CT chest images showing dense consolidation Left-hand side image: Axial chest CT showing the bilateral and extensive distribution of GGOs and consolidation. Right-hand side image: CT image displaying crazy-paving pattern (black arrow). Image source: Reference no. [7,18]

CT can provide visual insight into lung pathology in COVID-19 and respiratory manifestations such as pneumonia. The most common COVID-19 findings on CT are GGOs (Figure 6), dense consolidations (Figure 7 - left-hand side image) that block airways and blood vessels, enlargement of lung vasculature, and crazy-paving pattern (Figure 7 - right-hand side image), which is thickened interlobular septa superimposed on GGO [7,15,17]. Furthermore, GGOs and consolidations are typically bilateral in the peripheral aspect of the lungs (Figure 7), with predilection towards the posterior and lower lobes [17,22]. In severe cases the GGOs and consolidations are extensive and multifocal. Xiong et al., and Kaufman et al. describe that together they form a “white lung” appearance on CT. Consolidation findings in the lungs in COVID-19 patients can be quantified on the basis of the level of opacification and comparison of two consecutive CT scans [5,23]. 

In this study, the total opacification score in the upper, middle, and lower lobes calculated from the initial CT was significantly lower than in the seven-day follow-up CT (P< 0.001), indicating advancing involvement. Also, two strong correlations were found. First, before the patients received treatment and were admitted to the hospital, there was a positive correlation between the time interval in days from the onset of symptoms to initial CT, and total opacification score (R= 0.68, P< 0.01). Second, there was a strong association between high body temperature on initial presentation and the change in total score between initial and follow-up CT.

The risk that the severity of opacification would increase was 3.64 times greater in patients with high fever than those with normal temperatures. With age and sex adjustment, temperatures of 38.1°C to 39.0°C were positively associated with a four times higher increase in the total opacification score [4].

CO-RADS Scoring

CO-RADS scoring was developed for patients with moderate to severe symptoms commonly observed in COVID-19 to quantify the likelihood that the pulmonary involvement revealed by CT is due to COVID-19 [16]. CO-RADS categories range from 0 to 6. Category 0 implies a non-interpretable CT scan due to artifact; Category 1 implies very low suspicion and is probably attributable to non-infectious etiologies such as lung tumors or emphysema; Category 2 implies low suspicion if CT findings that point to infectious origin (e.g. tree-in-bud sign, centrilobular nodular pattern, lobar or segmental consolidation, lung cavitation) are not associated with COVID-19; Category 3 implies uncertainty if CT features associated with COVID-19 (e.g. peri-hilar GGO, homogenous extensive GGO with or without sparing of secondary lobules, or GGO with smooth interlobular septal thickening with or without pleural effusion) are also detected in other viral pneumonias; Category 4 implies high suspicion if CT features are typically seen in COVID-19 and in some viral pneumonias; Category 5 implies high suspicion if CT findings are classical for COVID-19, as described previously, and if one of the patterns aligns with the timeline of COVID-19 progression; Category 6 indicates proven COVID-19 if the RT-PCR test is positive. For inter-observer agreement, the Fleiss κ extracted by Prokop et al. [16] for all eight observers on CO-RADS was 0.47 (95% CI: 0.54, 0.62).

When the performance of CO-RADS was compared with those of RT-PCR and RT-PCR combined with clinical diagnosis, the average areas under the receiver operating characteristic curve (AUC) were 0.91 (95% CI: 0.85 0.97) and 0.95 (95% CI: 0.91, 0.99), respectively. In another retrospective study of 120 subjects done by Turcato et al. [24] the AUC of CO-RADS reported for patients with a positive RT-PCR, patients with a final clinical diagnosis of COVID-19, and patients with moderate to severe symptoms and a diagnosis of COVID-19, were 0.790 (95% CI: 0.704-0.876), 0.878 (95% CI: 0.812-0.943), and 0.890 (95% CI: 0.816-0.981), respectively. Prospective study - high negative predictive value (NPV) for CO-RADS less than or equal to 3.

**Figure 8:**
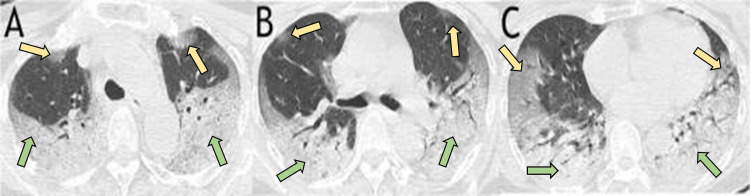
CT images showing various extents of consolidation and areas of GGO within both lungs The CO-RADS score was calculated as: for upper zone [A], 3 [consolidation] x 3 [50-75% distribution] x 2 [both right and left lungs] + 2 [ground glass opacity] x 1 [< 25% distribution] x 2 [both right and left lungs] For middle zone (B), 3 [consolidation] x 2 [25-50% distribution] x 2 [both right and left lungs] + 2 [ground glass opacity] x 2 [25-50% distribution] x 2 [both right and left lungs] For lower zone (C), 3 [consolidation] x (2 [25-50% distribution of the right lung] + 3 [50-75% distribution of the left lung]) + 2 [ground glass opacity] x (2 [25-50% distribution of the right lung] + 1 [< 25% distribution of the left lung]). Image source: Reference no. [16]

In the images below (Figure 8), CT images A, B, and C show various extents of consolidation and areas of GGO within both lungs based on the severity of the disease. The consolidations are pronounced in the posterior aspects of both lungs and the GGOs are apparent within the lung parenchyma anteriorly. The explanation for CO-RADS scores for each can be seen below the image. The areas of consolidation are indicated by green arrows and GGOs with yellow arrows.

Assessment of the Clinical Course/Progression of COVID-19 Based on CT Imaging Studies

The current literature describes the role of imaging in staging the progression of COVID-19. Jin et al. [25] distinguished five stages according to the progression of pathological features revealed by CT: ultra-early stage, early stage, rapid progression stage, consolidation stage, and dissipation stage. The ultra-early stage is 1-2 weeks after exposure to the virus and is asymptomatic. There is a single GGO or multiple patchy GGOs along with patchy consolidations and air bronchograms. The second (early) stage is 1-3 days after symptom onset. CT shows multiple scattered GGOs and interlobular septal thickening, with alveolar and interstitial exudates. The third (rapid progression) stage involves continued increases in exudates in alveoli and interstitium and vascular expansion. Light consolidation expands with inner air bronchograms. The fourth (consolidation) stage is 1-2 weeks after the onset of symptoms. Lung consolidations decrease in density and size on CT. Dissipation, the fifth stage, is 2-3 weeks after the appearance of symptoms. Consolidations become distinctly patchy while interlobular thickening with a grid-like pattern becomes more apparent. Yang et al. [26] also formed a clinical guideline based on symptom onset and response to the virus, distinguishing four stages (early, advanced, severe, dissipation), each with the characteristics previously mentioned. In the severe stage, CT scans show pleural thickening and effusion in addition to diffuse consolidations. There is bilateral involvement of patchy consolidation and grid-like thickening of interlobular septa.

**Figure 9:**
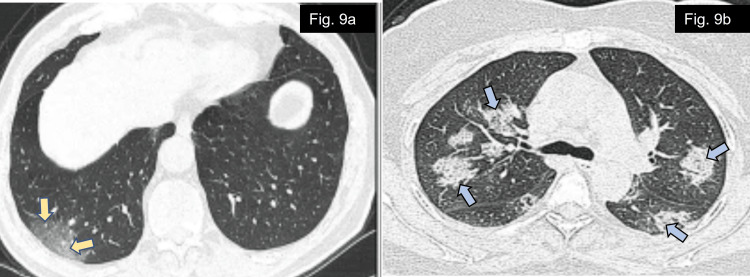
Area of consolidations within lungs of COVID-19-positive cases Image on the left (9a) showing an area of consolidation in the peripheral lung parenchyma of the right lung. Image (9b). Multiple areas of patchy consolidations bilaterally. Image source: Reference no. [25]

The images below show consolidations within the lungs of COVID-19-positive cases at various stages of onset (Figure 9). Figure 9a shows the ultra-early stage (1-2 weeks after exposure with no symptoms). GGO in the lower periphery of the right lung (indicated by yellow arrows). Figure 9b shows the early stage (1-3 days after symptom onset). Multiple, light, patchy consolidations (indicated by blue arrows) separated by thickened interlobular septae due to interstitial exudate in both lungs.

**Figure 10:**
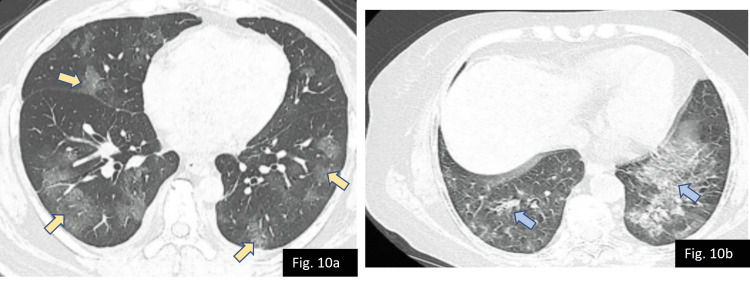
Findings within lungs at different stages of COVID-19 Image on the left (10a) shows the CT with yellow arrows pointing to areas of consolidation and in the image on the right (10b) they are indicated by a blue arrow in the CT. COVID-19: Coronavirus disease 2019 Image source: Reference no. [25]

The images below (Figure 10) show more findings at different stages of the disease. Figure 10a shows the rapid progression stage (3-7 days after symptom onset). Fibrous exudation connects the alveoli through the interalveolar space, forming a fusion. In both lungs, consolidations are larger, with air bronchograms. Figure 10b shows the consolidation stage (7-14 days after symptom onset). There are few lesions, and multiple patchy consolidations in the left lower lobe. There is bilateral involvement of patchy consolidation and grid-like thickening of interlobular septa.

**Figure 11:**
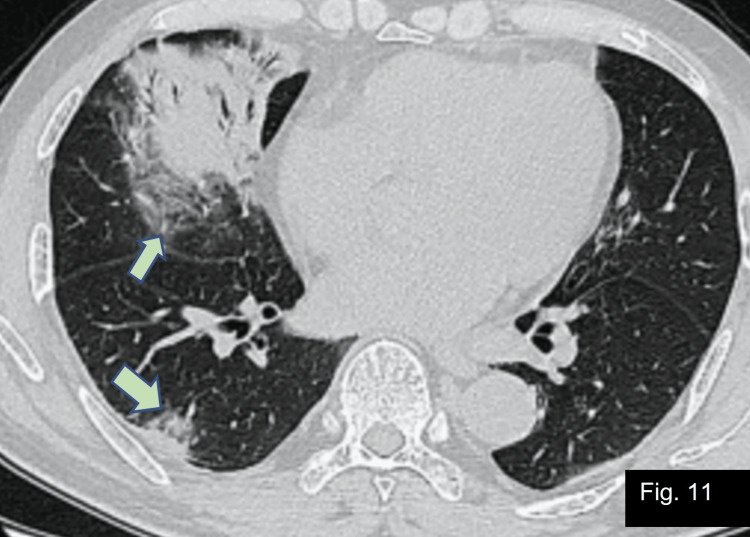
CT chest after 2-3 weeks of symptom onset Dissipation stage (2-3 weeks after symptom onset). There are very few lesions. Image source: Reference no. [25]

In the image below (Figure 11), the chest CT shows fewer areas of consolidation in the right lung, as the disease resolves over a period of weeks. The consolidated areas are indicated by green arrows. The left lungs look clear. 

Total lung involvement scores were also integrated into distinguishing stages for COVID-19. Zhou et al. [27] defined three stages of COVID-19 by measuring lung parenchymal involvement in over 272 individual CT scans from 100 patients according to the time from symptom onset: early rapid progressive stage (≤ 7 days), advanced stage (8-14 days), and absorption stage (>14 days). Following a clinical diagnostic guideline in China, the CT scans were stratified according to those three stages. The incidences of several CT signs were determined in each group to correlate changes in CT features with disease progression from the early rapid progressive stage to the absorption stage.

The results showed that the incidences of seven CT signs (GGO, GGO + consolidation, microvascular dilation, air bronchogram, subpleural transparent line, thickening of the pleura, and pleural retraction) showed a downward trend; the incidences of four CT signs (consolidation, GGO + reticular pattern, vacuolar sign, and pleural effusion) increased from the early rapid progressive stage to the advanced stage and decreased from the advanced stage to the absorption stage; and the incidences of three CT signs (subpleural line, bronchus distortion, and fibrotic strips) showed an upward trend. CT scan scores have been used as predictors for mortality and long-term effects of COVID-19. Yuan et al. [28] scored axial images by combining CT features with the involvement of lung parenchyma, which was divided into six zones. When the survival and mortality groups were compared, the CT score was significantly higher in the mortality group (30 vs. 12, respectively, P= 0.021). Han et al. [29] assigned CT severity scores to patients who recovered from severe COVID-19 by summing sub-scores based on parenchymal involvement (0, no involvement; 1, < 5%; 2, 5%-25%; 3, 26%-49%; 4, 50%-75%; 5, >75%) in five lung lobes. The authors found that a score ≥ 18 on the initial CT predicted the development of fibrotic lesions at a six-month follow-up CT (Odds ratio: 4.2, 95% CI: 1.2-14, p=0.02).

Radiographic Findings in the Diagnosis and Progression of COVID-19

The most common chest X-ray features of COVID-19 correlate well with those seen on CT and include consolidations and GGOs with a peripheral distribution and lower lobe predominance. Across studies, those features predominated between days 9 and 12 after symptom onset. Pleural effusions were seen rarely: 3% in a study by Wang et al.(2020) and <1% in a study by Goyal et al. [30,31].

The articles reviewed showed that as few as 20% of patients (no control group, so no true sensitivity value) demonstrated a typical COVID-19 presentation on chest X-ray with up to 89% sensitivity. It is notable that the sensitivity of this modality tended to increase throughout the pandemic, in areas with high disease prevalence, and with severity of disease [31].

A study by Goyal on 422 patients graded the severity of disease on a 1 - 8 scale and found that the average X-ray score was 2.8/8 for patients with mild symptoms and 4.2/8 for those with moderately severe symptoms. Based on their grading system, they considered any patient with a score less than 3 as mild and needing only symptomatic treatment. However, 93% of patients with a score of 4 or above required assisted ventilation. This study therefore set an X-ray severity score of 4 as the baseline for critical care management. Mingli et al. described the CT changes in disease progression in the patients below [16].

**Figure 12:**
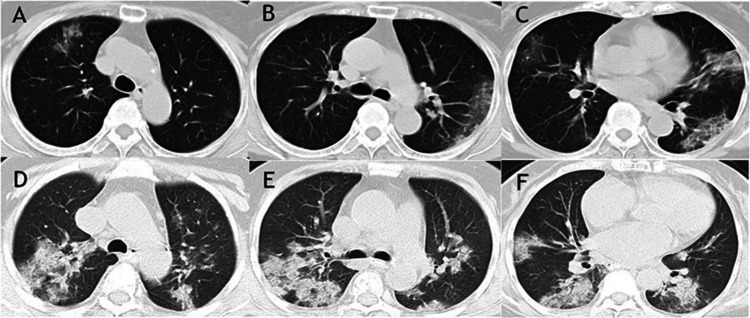
Pulmonary changes with progression of the disease A-C: stages of the disease that resulted in resolution. D-F: CT images of a patient who succumbed to the disease. Image source: Reference no. [16]

In the image below (Figure 12), there is clear demonstration of the progression of the disease in two cases. A-C indicate the pulmonary changes seen in a chest CT of a 76-year-old woman who survived the disease; D-F show progressive changes in a 72-year-old woman who succumbed to it.

Lung Ultrasound in the Diagnosis of COVID-19

Three articles reported sensitivity and specificity values for LUS. A systematic review of imaging in the diagnosis of COVID-19 by Islam et al. [29] reported a sensitivity of 96.8% and a specificity of 62.3%. Another systematic review by Stogiannos et al. [32] reported a sensitivity of 93% and a specificity of 95%. Independent research by Narinx et al. [33] on the feasibility of LUS reported a sensitivity of 93.3% and a specificity of 21.3%.

Despite the lack of guidelines for COVID-19 screening with LUS, most studies have used point-of-care ultrasound. A study of LUS findings in patients with COVID-19 pneumonia showed a glass rocket appearance, pleural line thickening, and confluent B lines in all patients. B lines are white, vertical lines extending from the pleura, and a glass rocket appearance denotes five or more B lines. Half the patients in the study also exhibited subpleural consolidations [4]. Another study by Deng et al. [34] reported crowded B-lines and multifocal consolidations as the most common LUS findings in COVID-19 patients. This same study found high consistency between LUS and CT scores in the follow-up of critically ill COVID-19 patients.

In one literature review, LUS had a relatively high diagnostic value in asymptomatic COVID-19 patients [29]. Other reported benefits of LUS include portability, reduced exposure risk, and suitability for pregnant women. Two studies noted a lower risk of exposure associated with LUS than other radiological interventions. The same two studies also noted the importance of LUS in the management of pregnant women because of the absence of radiation [9,29].

Discussion

Chest CT

The diagnosis and appropriate management of COVID-19 are crucial steps in providing the most efficient medical care for both the patient and the overall community during the pandemic. Despite the relatively high specificity of 99.5% in RT-PCR, low sensitivities such as 56% and 71% can produce false-negative results that will contribute to the continued spread of SARS-CoV-2. Likely explanations for this include a lack of robustness in the technology, low viral load in the sample, or inadequate clinical sampling [9,18]. Chest CT is a noninvasive, rapid modality that offers high sensitivities up to 98% in the detection of COVID-19. Ai et al. reported that among 413 patients with suspected severe acute respiratory syndrome coronavirus 2 infection who tested negative on RT-PCR, 308 (75%) showed positive chest CT findings [19]. About 48% of the 308 patients were deemed highly likely cases and 33% as probable cases. Although there is an overlap in CT findings between COVID-19 pneumonia and other viral pneumonias such as influenza and adenovirus, the high level of sensitivity was crucial during the growing epidemic as it transitioned into a pandemic [35,36]. Isolating all patients with positive CT findings would pose a lower risk of spreading the virus than ruling out COVID-19 in patients with typical symptoms and a potentially false-negative RT-PCR result. Another limitation of CT is the lower sensitivity for diagnosing COVID-19 in patients who are asymptomatic or in the early stage of the disease [1]. For example, Salehi-Pourmehr et al. [21] provided a two-pronged explanation that the low sensitivity of 64% could be attributed to subjects having chest CT scans on the day of hospital admission while they were in the beginning phase of the disease. Therefore, the sensitivities determined by Fang et al. and Ai et al. (98% and 97%, respectively) could be biased because the patient populations enrolled presented with symptoms of acute respiratory syndrome [18,19].

The overall findings suggest that CT is not currently suitable as a first-line tool for screening out COVID-19, but must serve as a supplement or an alternative in case of a suspected false-negative RT-PCR.

Chest CT can help radiologists further by providing a visual assessment of the abnormalities in a patient’s lungs infected with SARS-CoV-2. The studies selected across the literature search consistently indicated typical features of COVID-19 on CT: bilateral GGOs, consolidations, and crazy paving patterns in the lung periphery and commonly in the posterior segments of the lobes [3,7,15,22,37]. The rapidity of CT means that in emergency situations, patients who present with characteristic CT findings of COVID-19 and are awaiting an RT-PCR result can be quickly isolated and appropriately managed [17]. Quantifying the severity of lung lesions on CT also brings value to the clinical management of patients with COVID-19. Xiong et al. [23] first found an increase in lung involvement, based on the total opacification score, between the initial and follow-up CT scans. Next, there was a significant positive correlation between the number of days from beginning of symptoms to the initial CT and the total score before treatment and hospital admission. This result suggests that the time between symptom onset and the initial CT scan critically influence the level of medical care in COVID-19 infection. Finally, the significant positive correlation between high fever on initial presentation and the change in total score between initial CT and follow-up CT supported increased body temperature in COVID-19 as a risk factor for adverse CT outcomes. Therefore, a high body temperature necessitates careful monitoring for advancement of lung abnormalities that will lead to adjustment in patient management.

One limitation is the small sample size. This calls for more investigations with more patients, which will give a clearer indication of the value of CT. A disadvantage of CT, as previously mentioned, is that it can yield false negatives in the early stages of the disease. In the review papers and primary literature extracted from the search, the images and CT findings described only represented patients with symptoms of COVID-19. Further study seems desirable to assess the careful monitoring of CT findings in asymptomatic patients to detect and manage COVID-19 efficiently.

CT and COVID-19 Progression

Chest CT can enable clinicians to be vigilant of the progression of lung pathology in COVID-19. By using staging methods as developed by Jin et al. and Yang et al., in combination with the patient’s risk factors, physicians could predict the onset of severe complications [25,26]. This result suggests a need for further study of the correlation between stages based on CT imaging and the progression of symptoms. Zhou et al. presented changes in CT signs over the clinical course of COVID-19 by retrospectively comparing incidences of thirteen signs in three groups (early rapid progressive stage, advanced stage, and absorption stage) [27]. Given that the intensity of the CT signs was greater in the advanced stage (e.g., GGO + reticular pattern, vacuolar sign, consolidation) than the rapid progressive stage (e.g., GGO, pleural thickening and retraction), it can be inferred that lung parenchymal pathology worsened in disease progression. In addition, the intensity of the signs decreased into the absorption stage while the incidence of repair indicators (e.g., subpleural line, bronchus distortion, fibrotic strips) increased. This could have suggested improvement in lung pathology over time. The authors noted that despite this general pattern, some patients had individual trajectories as their lung abnormalities were partially progressive and partially absorbed. This indicates that radiologists must be aware of case-by-case monitoring for each patient with COVID-19 in addition to the classical findings and changes seen on CT. Continued focus on consistent trends in CT features of COVID-19 while interpreting the diversity of CT results in relation to these trends could further unpack the complex disease course of COVID-19.

There also needs to be a standard staging system that can limit subjectivity after analyzing the CT findings. Scoring the severity of the CT image can be helpful in predicting the progression of COVID-19. As noted by Yuan et al., the CT severity score was significantly higher in the mortality group than the survival group [28]. Han et al. demonstrated that through their scoring system, a cutoff score ≥ 18 based on parenchymal involvement could predict fibrotic abnormalities in a six-month follow-up CT. Based on these findings, a relatively high CT score can guide the medical team toward the most appropriate treatment and timeline for follow-up imaging. However, the sample sizes in these investigations were small, and the two studies used different scoring methods. Additional studies must enroll larger sample sizes and use consistent scoring methods [29].

X-Ray

The consolidations and GGOs seen in COVID-19 pneumonia are airspace opacities, and these are not seen as well on chest X-rays as on CT. A review by Sanchez-Oro et al. [38] proposes that this could be the underlying reason why chest X-ray has lower sensitivity than CT to COVID-19 pneumonia. They found that parenchymal changes are most severe 6-11 days after the onset of symptoms, and this correlates well with Goyal finding the most severe chest X-ray findings during days 9-11 and Wong et al. during days 10-12. Goyal et al. conclude that their institution performing chest X-rays earlier in the course of the disease while the airspace opacities were still too subtle for X-ray to detect could explain why his study, with only 20% of patients demonstrating typical COVID-19 pneumonia, correlated with Wong’s study demonstrating a sensitivity of 69% [31].

While CT remains the first line and best choice for imaging suspected COVID-19, chest X-ray remains a valuable resource when other options are limited or unavailable. Furthermore, a radiologist’s ability to detect COVID-19 on chest X-ray can further support finding incidental cases of COVID-19, and the grading system produced by Goyal et al. is useful for predicting the care and management required for each patient [31].

Lung Ultrasound

This literature review included 10 articles discussing LUS in the diagnosis and management of COVID-19. The systematic review by Islam et al. warned that quantitative values should be interpreted with caution. A formal assessment of the quality of articles included in the review revealed high or unclear risk of bias in most studies. The sensitivity and specificity of LUS for diagnosing COVID-19 in a study that involved 100 participants were 96.8% and 62.3%, respectively [39]. A systematic review by Stogiannos et al. was also consulted but it did not formally assess the quality of the articles included [30]. The study by Narinx et al. comprised a relatively small sample and included only patients admitted to the emergency department. Owing to the wide range of reported sensitivity and specificity values for LUS, along with the high risk of bias in the studies consulted, we do not consider these values reliable [32,33].

**Figure 13:**
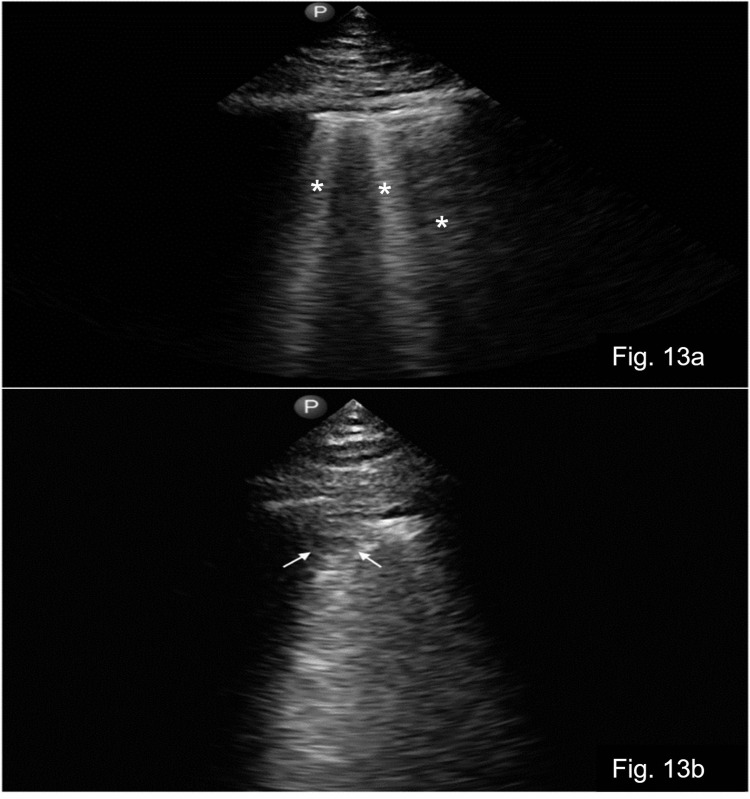
Lung ultrasound images 13a: Confluent B lines on lung ultrasound indicating fluid within the lung. 13b: White arrows indicating subpleural consolidations on lung ultrasound.

In the two studies that examined LUS findings in COVID-19 patients, the most common features included confluent B-lines (Figure 13). B-lines in ultrasound result due to discreet vertical reverberation artifacts that originate from the pleural line. These can extend to the depth of the image without decreasing in intensity and they tend to move synchronously with lung sliding. Due to their appearance these are commonly referred to as ‘lung comets’ or ‘glass rockets’ (Figure 13a). This finding might indicate pleural effusion with accompanying atelectasis. Multifocal consolidations (Figure 13b) indicate airspace opacification. Described as a shred sign or fractal sign, is indicated by static images of subpleural hypoechoic regions that have irregular (shredded) deep borders (fractal line), normally abutting aerated lung that has echogenic artifacts.

In the two studies that examined LUS findings in COVID-19 patients, the most common features included confluent B lines (Figure 13a) and multifocal consolidations (Figure 13b). Yasukawa and Minami [40] studied a small sample of 10 patients; however, their results were consistent with previous studies. Deng et al. examined a relatively large sample of 72 patients; however, not all the subjects who underwent LUS were included [34]. Therefore, the results did not necessarily reflect the entire study population.

The benefits of investigations such as POCUS (point-of-care ultrasound) includes accessibility, portability, cost-efficiency, and it can be done by trained personnel where the patients are being treated. Based on necessity, this procedure may be performed by emergency room physicians, internists or by nurse practitioners. A disadvantage of this is that the interpretation of the findings may vary based on the level of training and availability of radiologists. Additionally, LUS reduces the risk of exposure between patients and healthcare professionals; therefore, its use could help mitigate the spread of COVID-19. Because it produces no radiation, LUS is also suitable for managing pregnant women with COVID-19. A significant disadvantage of LUS is the low reported specificity values [41]. Also, aerated lungs during LUS could prevent the detection of deeper lesions. For these reasons, the authors suggest that LUS should complement the management of patients with COVID-19, particularly pregnant women, but the findings should be confirmed by RT-PCR or chest CT. 

Limitations

This research is subject to several limitations. First, most of the studies examined relatively small samples. As mentioned previously, the WHO has reported about 180 million cases of COVID-19; most studies had sample sizes of less than 50 patients, extremely small compared to the overwhelming number of patients affected by COVID-19. This could compromise the reliability and external validity of the results. Additionally, the authors of this review did not assess study quality formally, and only three of the review articles cited included such formal assessments. The QUADAS-2 assessment in these three articles revealed high or unclear risk of bias in most of the studies included in their respective literature reviews. It is also possible that the sensitivity and specificity of radiological methods could be overestimated owing to publication bias and bias towards symptomatic patients. Further research is needed to understand how the various radiological methods perform against RT-PCR testing on asymptomatic patients. Another limitation that could affect the reported sensitivities and specificities of the radiological methods is that most of the studies used RT-PCR as the reference standard; however, RT-PCR has imperfect sensitivity and a significant false-negative rate. Finally, it is worth noting that all the studies were conducted over a period of two months, particularly the early months of the pandemic from January to May 2020, and therefore represent only a snapshot in time of the current research.

## Conclusions

This literature review supports the opinion that CT is the most reliable radiological tool for diagnosing and managing patients based on the progression of a COVID-19 infection. CT is especially indicated in conjunction with RT-PCR, in part because RT-PCR yields a significant percentage of false negative test results. With CT, physicians could pinpoint the onset and progression of the disease and administer appropriate medical interventions. The review indicates that chest X-rays and ultrasound serve as valuable technological resources in COVID-19 diagnosis and management, especially when CT is unavailable or contraindicated. Having analyzed the gaps and weaknesses, such as limited sample size and accessibility, as mentioned in these articles, it will be important for clinicians to analyze emerging studies and data to determine which radiological investigation is best suited to diagnosing and managing COVID-19 patients.
